# Interaction Networks Converging on Immunosuppressive Roles of Granzyme B: Special Niches Within the Tumor Microenvironment

**DOI:** 10.3389/fimmu.2021.670324

**Published:** 2021-04-01

**Authors:** Weinan Wang, Rui Zou, Ye Qiu, Jishuang Liu, Yu Xin, Tianzhu He, Zhidong Qiu

**Affiliations:** ^1^ School of Pharmaceutical Sciences, Changchun University of Chinese Medicine, Changchun, China; ^2^ School of Basic Medical Sciences, Changchun University of Chinese Medicine, Changchun, China

**Keywords:** granzyme B, immunosuppression, tumor microenvironment, suppressor cells, regulation networks

## Abstract

Granzyme B is a renowned effector molecule primarily utilized by CTLs and NK cells against ill-defined and/or transformed cells during immunosurveillance. The overall expression of granzyme B within tumor microenvironment has been well-established as a prognostic marker indicative of priming immunity for a long time. Until recent years, increasing immunosuppressive effects of granzyme B are unveiled in the setting of different immunological context. The accumulative evidence confounded the roles of granzyme B in immune responses, thereby arousing great interests in characterizing detailed feature of granzyme B-positive niche. In this paper, the granzyme B-related regulatory effects of major suppressor cells as well as the tumor microenvironment that defines such functionalities were longitudinally summarized and discussed. Multiplex networks were built upon the interactions among different transcriptional factors, cytokines, and chemokines that regarded to the initiation and regulation of granzyme B-mediated immunosuppression. The conclusions and prospect may facilitate better interpretations of the clinical significance of granzyme B, guiding the rational development of therapeutic regimen and diagnostic probes for anti-tumor purposes.

## Introduction

Granzyme B (GrB) is a serine protease famous for its activity in proteolysis-mediated apoptosis and works as a critical effector molecule of cytotoxic lymphocytes (CLs) against pathogens during immunosurveillance (1). Upon being properly activated, CLs could recognize the ill-defined cells and secrete cytotoxic granules into an immunological synapse where granzyme B is endocytosed into the cytosol of target cells and triggers the downstream apoptotic pathways (2).

For a long time, granzyme B has been well-accepted as a representative marker for the priming of immunity and efficient killing of tumor cells. In light of the anti-tumor reputation of granzyme B, the development of GrB-based/targeted theranostics has been advanced rapidly in recent years (3, 4). However, the expression of granzyme B is not always positively correlated with anti-tumor performance. Some researchers even noticed that GrB-deficient mice demonstrated better eradication ability of either allogeneic or syngeneic tumor cells than did wild-type mice (5). Although granzyme B is selective on conserved amino acid sequences of its substrates, its cytotoxicity is non-specific to tumor cells, suggesting that granzyme B in active form, especially the one released to extracellular space, might harm both parties of the immune responses within the tumor microenvironment (TME) (5). These observations and hypothesis bring up a question: is granzyme B always a noteworthy ally against tumors or a waverer that sometimes works in the opposite way.

**Figure 1:**
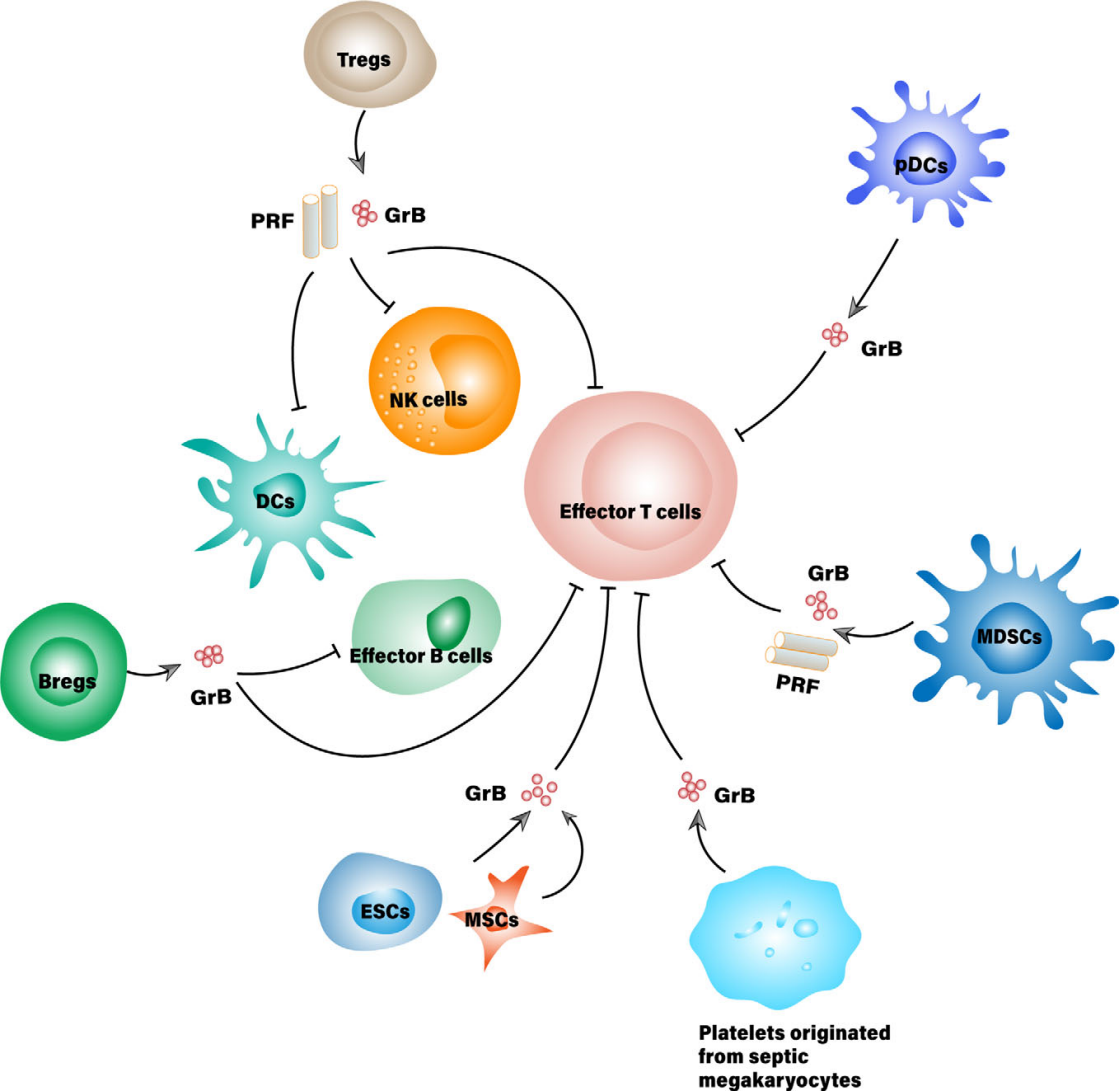
Suppressor cells that secrete granzyme B for immunosuppressive purposes. MDSCs, Myeloid-derived suppressor cell; ESCs, Embryonic stem cell; MSCs, Mesenchymal stem cells; PRF, Perforin.

Increasing evidence has emerged to support the pleiotropic roles of granzyme B within which the immunosuppressive effects being highlighted. Aside from CD4^+^/CD8^+^ T cells and NK cells, the expression of active granzyme B is observed in many other types of cells such as B cells, dendritic cells, macrophages, mast cells, basophils, keratinocytes and chondrocytes etc., some are even the bystanders of lymphocytes (6). One part of them constitutively expresses granzyme B, while the other part only expresses it under proper stimulations. The significance of granzyme B expressed by these cells lies in not only their intrinsic feature but also the context that defines their roles. So far, several cell types, exemplified by T regulatory cells (Tregs), B regulatory cells (Bregs), and plasmacytoid dendritic cells (pDCs) are discovered to secrete granzyme B for immunosuppressive purposes as demonstrated in **Figure 1**, though the regulation networks are yet to be established (5, 7–9).

The immunosuppressive role of granzyme B was initially observed in the degradation of T cell receptor (TCR) zeta chain that is essential to the surface expression of TCRs for T cell development (10). The loss of TCR zeta chain seems to be a common phenomenon in tumor-infiltrating lymphocytes, especially the ones suffering from immune exhaustion. Although several mechanisms might involve the loss of TCR zeta chain, the degradation caused by granzyme B practically linked granzyme B with the immunosuppressive components in TME. Nevertheless, the degradation of TCR zeta chain is not the only way in which granzyme B suppresses the priming immunity. More mechanisms and participants have been unearthed in association with immunosuppressive roles of granzyme B, summing up a clearer picture of the GrB-expressing niches in TME, which we are going to discuss detailly in this review.

## GrB^+^ Suppressor Cells

### T Regulatory Cells

Tregs are a suppressive subset of T cells with the typical hallmarks of CD25 and Foxp3 expression. Although Tregs only occupy a small proportion of CD4^+^ T cells, they play central roles within the whole immunosuppressive microenvironment either in healthy or ill-defined conditions (11). They are essential to maintaining peripheral tolerance and immune homeostasis in the setting of autoimmune diseases while suppressing beneficial anti-tumor immunity in TME to facilitate tumor evasion and metastasis.

Varied stimulatory molecules induce the differentiation of Tregs into diversified subsets, thereby exerting their immunosuppressive functions against different immune cells *via* multiple pathways (12). For instance, Tregs competitively consume interleukin 2 (IL-2) with weakly activated T effector cells (Teff), causing the suppression of adaptive immune responses (13). Moreover, Tregs secrete IL-10, IL-35, adenosine, and transforming growth factor-β (TGF-β) while express surface molecules, such as cytotoxic T-lymphocyte antigen 4 (CTLA-4), lymphocyte activation gene 3 (LAG-3), and programmed death-1 (PD-1) for general immunosuppressive purposes (14). Other than inhibiting cell function or decreasing cell viability, Tregs can directly induce apoptosis or cytolysis of B cells, antigen-presenting cells (APCs) and Teff, etc., through a GrB-mediated manner (15, 16). This immunosuppression pattern may or may not require cell-to-cell contact, indicating different mechanisms that trigger granzyme B attack.

The association between granzyme B and immunosuppressive effects in Tregs was initially established based on the frequent presence of GrB^+^ Tregs in malignant tumor lesions (17). Also, high levels of GrB^+^ Treg was found to negatively correlated with the occurrence of acute graft-versus-host disease after hematopoietic stem cell transplantation, implying a regulatory effect on active participants of adaptive immunity (18). The expression and secretion of granzyme B by Tregs seem to be context-dependent, as evidenced by the disproportionate level of granzyme B in naturally occurring Tregs (nTregs) from thymus comparing to stimuli-inducible Tregs (iTregs) in TME (19).

CD4^+^CD25^+^FoxP3^+^ Treg is a typical phenotype that bears granzyme B for immunosuppressive purposes. In contrast to CD4^+^CD25^+^ Tregs, they present an elevated expression of CD275 (ICOSL), CD278 (ICOS), major histocompatibility complex (MHC) II and loss of CD73, which could suppress primed T cells *in vivo via* a GrB-dependent way (5, 20, 21). Unlike tumor-infiltrating Tregs, the circulating Tregs demonstrate very few GrB^+^ cells with frequencies of lower than 0.3% in all subtypes, further highlights the latent stimuli in specific niches that determine the presence of GrB^+^ Tregs (15).

Generally, the expression of granzyme B in T cells can be activated by prolonged TCR stimulation through CD3/CD28. However, the generation of GrB^+^ Tregs needs the participation of IL-2, as either TCR stimulation or IL-2 treatment alone would fail to induce granzyme B in Tregs (22). In addition to CD3/CD28, stimulation of naive CD4^+^ T cells with anti-CD46 monoclonal antibodies could convert them into granzymes/perforin/IL-10 producing Tregs that kill allogeneic cells as well as autologous immune cells (23, 24).

The induction of other immunosuppressive molecules often accompanies the generation of granzyme B in Tregs. Latency-associated peptide (LAP), the N-terminal pro-peptide of the TGF-β precursor, could facilitate the conversion of naive Tregs to iTregs. Studies have shown that these iTregs expressed more granzyme B and TGF-β than their LAP negative counterpart, exerting their immunosuppressive effects *via* both granzyme B and TGF-β mediated mechanisms (25–28). In another case, the up-regulation of granzyme B was observed in a “self-feeding” process of Tregs caused by an intercellular CC motif ligand (CCL) 1-CC chemokine receptor (CCR) 8 interaction, leading to synchronized up-regulation of FoxP3, CD39 and IL-10, which substantiated the *in vivo* proliferation and immunosuppressive activities of these Tregs (29). Even when encountered with OX40 agonist, potential immunotherapy that enhances anti-tumor immune responses, it did not harm the regulatory ability of Tregs due to the simultaneous increase in granzyme B, IFN-γ, and T-bet expression.

Although the immunosuppressive ability of Tregs would sometimes be reprogrammed or overwhelmed by a subtle environment, the expression and secretion of active granzyme B in Tregs could be a valuable prognostic for immunosuppressive status (30).

### B Regulatory Cells

B cells have been classically associated with antibody secretion, antigen presentation, and T cell activation. However, the presence of B cell-mediated immune response does not always positively correlate with a benign prognosis during anti-tumor therapy. Some subsets of B cells, particularly the ones from tumor-derived lymph node (TDLN), exhibit regulatory phenotype and inhibitory activity toward other anti-tumor participants, probably contributing to the immunological tolerance of malignancies (31). These B cells with regulatory effects are termed as Bregs though there are no consensus markers about this classification (32).

The typical phenotype that different Bregs share is the secretion of IL-10 and expression of CD1d and CD5, although subsets of Bregs are known to express not only IL-10 but also other inhibitory molecules, including PD-L1, granzyme B, and TGF-β. Bregs express these cytokines for specific reasons. For instance, TGF-β from Bregs could induce iTregs which would, in turn, facilitate the differentiation of immature B cells into Bregs, hence synergistically controlling the inflammatory responses (33). GrB^+^ Breg is a special and potent regulatory subtype phenotypically and functionally distinct from IL-10-producing Bregs (B10 cells) in humans. In human GrB^+^ Bregs, most of the regulatory molecules are expressed primarily on GrB^+^, but not GrB^-^ B cells. This suggests that granzyme B might be an important novel marker indicative of immunosuppressive effects of human Bregs (34). IL-21 derived from CD4^+^ T cells was found to dominantly drive the generation of GrB^+^ B cells, during which CD40L was identified as an important determinant for the differentiation of B cells into either plasma cells or GrB^+^ B cells (35). Only when cultured with IL-21^+^ CD40L^-^ Th cells would B cells directly differentiate into GrB^+^ Bregs (36). The population of GrB^+^ Bregs is also positively correlated with IL-21 production. B cells from tolerant recipients but no other patients could regulate both the number of IL-21^+^ T cells and IL-21 production, suggesting a feedback loop that increases excessive B cell activation and endows the regulatory ability (37). Subsequently, GrB^+^ Bregs potently suppress the proliferation of co-cultured CD4^+^ T cells in a GrB-dependent manner. Aside from IL-21 producing cells such as CD4^+^ T cells, follicular helper T (Tfh) cells, and Natural killer T (NKT) cells, GrB^+^ Bregs also target excessive B cells for self-regulatory purposes as well as other bystander immune cells *via* paracrine mechanisms (7).

GrB^+^ B cells were unveiled to have pleiotropic roles in immune responses. One is the regulatory role that could maintain allospecific tolerance, and the other is the effector role against infected or ill-defined intruders (38). Within peripheral circulation, B cells from healthy individuals could produce and secrete granzyme B while encountering sufficient IL-21 and the stimulation of B cell receptors. A higher frequency of GrB^+^ B cells in peripheral blood often correlated with immune tolerance in the settings of autoimmune diseases, viral infection, and tumor progression (39). On the other hand, some GrB^+^ B cells were evidenced to initiate an attack against tumor cells due to its MHC-independent recognition of antigens. Such phenomenon often occurred in the early stage of neoplastic process, and, as the oncogenesis progressed, GrB^+^ B cells were gradually polarized into Bregs that might lead to malignancies during late-stage cancer (7, 38). That explains why GrB^+^ B cells found within the microenvironment of different tumor types were usually associated with the progress and metastasis of tumors.

The immunosuppressive mechanism exerted by GrB^+^ Bregs mainly converged on the GrB-dependent degradation of T cell receptor zeta-chain, which is similar to that by Tregs and pDCs. However, the ways Bregs work on other immunological participants for suppressive goals, especially those independent of T cell receptors for activation, still remain obscure (40, 41). In addition to direct inhibition of effector cells, Bregs with activated STAT3 are found in proximity to tumor vasculature and proved to be proangiogenic and positively correlated with tumor progression. Considering that STAT3 is a critical upstream transcription factor for granzyme B expression, such tumorigenic effects of B cells might partially attribute to either the cytotoxicity of granzyme B toward ambient effector cells or the proteolysis of extracellular matrix (ECM) by granzyme B (42).

Some researchers have tried to decipher the phenotypic signature of Bregs that could signify the expression level of GrB, leading to a few meaningful results as presented in **Table 1** (33, 34). Nevertheless, puzzles delineating the phenotypes of GrB^+^ Bregs are yet to be settled. The ambiguity might relate to the origin of B cells which confer different phenotypes to Bregs in TDLNs, peripheral blood, and tonsil (43).

**Table 1:** General information of GrB^+^ Bregs documented.

No.	Phenotype	Origin	Key regulatory molecules	Disease model
1	CD19^+^CD38^+^CD1d^+^CD14^+^IgM^+^	Human	CD25, IDO, IL-10, GrB	Epithelial cancers (breast,cervical, ovarian, colorectal, and prostate carcinoma)
2	CD19^+^CD5^+^CD43^+^CD86^+^CD147^+^	Human	GrB	HIV-1
3	CD19^+^CD5^+^CD27^+^CD138^+^CD38^+^	Human	GrB	Kidney transplant

### Plasmacytoid Dendritic Cells

Dendritic cells comprise versatile subsets designated to carry out different missions in response to immunologic stimuli. Some of them are determined effector cells against pathogen while others exert pleiotropic effects under different circumstances (44). Plasmacytoid dendritic cells (pDCs) play a crucial role during innate immunity by secreting bulk amounts of type I interferons (IFNs) in response to Toll-like receptor (TLR)–mediated pathogen recognition. Besides, pDCs can contribute to adaptive anti-tumor immunity by activation of antigen-specific T cells (45).

However, the presence of pDCs is not always beneficial to the boost of immunities. It has been evidenced in some cases that the complex interaction of pDCs with tumor cells and their microenvironment might lead to immunologic tolerance (46). For instance, factors such as TNF-α, TGF-β and IL-10 would abrogate the anti-tumor responses from pDCs and facilitate their pro-tumorigenic effects (47). The immunosuppressive roles of pDCs are closely associated with the functionality of Tregs because pDCs are one of the main driving forces for the development of Tregs in T-lymphocyte-rich areas of lymphatic organs (48). Hence an increase in intratumoral pDCs was often observed with simultaneous increase of Foxp3+ regulatory T cells in the same lesion and positively correlated with tumor vascular density (49). In a resting state, pDCs might induce unbiased Th1, Th2, or Treg responses, whereas, upon being activated with CD40 ligand (CD40L) and interleukin-3, pDCs specific of ICOS ligand (ICOSL) expression preferentially enhanced the generation of IL-10-secreting nTregs in periphery blood (47, 50). Such CD40-CD40L-mediated interaction between pDCs and nTregs established a feedback loop critical to pDC maturation and nTreg differentiation in the steady-state human thymus (51). In addition to the indirect immunosuppressive effect relating to Tregs, pDCs could directly participate in the immunomodulatory process *via* autocrine and/or paracrine mechanisms, such as *via* the secretion of IDO, ICOSL and Granzyme B, etc (8, 52, 53). Unlike effector T cells and NK cells which express and secrete perforin and granzyme B synergistically to fight against cancer, pDCs can produce and utilize a bulk amount of granzyme B independent of perforin. pDCs secrete granzyme B to the extracellular area, where it plays dual roles for anti-tumor immunity as it would help process peptide antigen to facilitate cross-presentation while generally suppress T cell activation and expansion through degrading the zeta chain of its TCR (54, 55).

Some researchers thought only specific subtypes of pDCs highly express and secrete granzyme B, as evidenced in a squamous carcinoma model (56). Another well-accepted notion suggested that the production of granzyme B could be induced and promoted in pDC precursors by certain immunosuppressive cytokines, including IL-3, IL-10, and IL-21. IL-3 was proved to be pivotal to GrB induction in pDCs (8). Literature has reported that IL-3 stimulated pDCs were able to decrease the population of both CD4^+^ and CD8^+^ T cells in a GrB-dependent manner. Such immunosuppressive effect of pDCs was further enhanced by IL-10, probably due to its contribution to granzyme B production, but inhibited by TLR stimulation which would downmodulate granzyme B expression in pDCs and polarize them into tumoricidal phenotypes (34, 57). IL-21 is a pleiotropic cytokine with a broad range of actions converging on immunogenicity (58). However, it could also induce the expression and secretion of granzyme B in pDCs, which is partially responsible for the pDC-mediated downregulation of CD4^+^ T cell proliferation (45). The regulatory side of IL-21 induced pDC can be reversed by the autocrine of type I IFNs which is consistent with the observation that TLR stimulation would convert GrB^high^ pDCs into its GrB^low^ counterpart with immunogenic feature (59).

### Other Cell Types

Expression of granzyme B is not specific to cytotoxic lymphocytes as many other cell types have been proved to express and secrete granzyme B under defined circumstances.

Except for conventional participants in immune responses (dendritic cells, macrophages, myeloid-derived suppressor cells, mast cells, basophils and B cells, etc.), these GrB expressing cells also include non-lymphocytes such as keratinocytes, platelets, human articular chondrocytes, and even cancer cells (60). Some cell types express GrB with perforin and other members of the granzyme family, which are often regarded as effector cells against cancer, while others express GrB independent of those cytotoxic components that might lead to pleiotropic effects (6).

Myeloid-derived suppressor cells (MDSCs) are one of the critical immunosuppressive cells against effector T cells, NK cells, dendritic cells and macrophages in the TME (61, 62). Even though their mechanisms of action are yet to be established, the clinical and experimental practice has demonstrated that tumors densely infiltrated with MDSCs are associated with poor prognosis and resistance to immunotherapies (63). Previous studies unearthed the metabolism of L-arginine and the generation of excessive ROS as major strategies that MDSCs invited to suppress immunological responses (64, 65). In recent years, some researchers had noticed a contact-dependent suppression of T lymphocytes by MDSCs and linked such phenomenon to the way cytotoxic T cells kill their targets *via* granzyme B/perforin (66). Then the expression of perforin and Granzyme B was validated in *in vitro* model of MDSC culture, ex vivo experiments of MDSCs isolated from tumor-bearing mice, and MDSCs from human. After deleting perforin/GzmB in MDSCs *in vivo*, an increased amount of CD8+ T cells appeared in the tumor lesion together with better therapeutic performance, suggesting an immunosuppressive role of granzyme B from MDSCs. Nevertheless, the detailed interaction between Granzyme B in MDSCs and the promotion of tumor growth still keep in the dark and warrant further investigation (9).

Sometimes seemingly innocent bystanders in body fluid could be educated into “granzyme B-armed killers” toward active lymphocytes. As in the case of sepsis, platelets were found accumulating in lymphoid microvasculature and suspicious of contributing to sepsis-related lymphoid apoptosis. Granzyme B, independent of perforin, secreted by these platelets, was a prerequisite to the lymphodepletion process, which required cell-to-cell contact with healthy lymphocytes. The immunosuppressive roles of GrB^+^ platelets were further substantiated in either the *in vivo* experiment that the absence of granzyme B slows sepsis progression or the ex vivo proof that platelets from septic mice radically decrease the population of healthy splenocytes through GrB-induced apoptosis (67, 68). Such unique platelets originated from septic megakaryocytes with an upregulated *Itga2b* gene which altered the mRNA profiles of the platelets and empowers them with the functions of granzyme B (69).

Embryonic stem cells (ESCs) and mesenchymal stem cells (MSCs) are long known to possess immunosuppressive potential, though the mechanisms are still unclear. ESCs could increase the proportion of FoxP3^+^ Tregs during alloimmunity as well as direct their regulatory effects toward CD4^+^ T cells through expression and secretion of granzyme B. The immunosuppressive process mediated by these stem cells requires cell-to-cell contact and is independent of perforin, PDL-1, or Fas ligand, etc. (70). While in the case of MSCs, the situation is more complicated and debatable. MSCs freshly isolated from healthy donor bone marrow were found to express and secrete a bulk amount of enzymatically active granzyme B, which was initially hypothesized to be a major suppressive molecule. Nonetheless unambiguous immunosuppression occurred in a co-culture of MSCs and CD4^+^ T cells, researchers failed to validate the immunosuppressive roles of granzyme B by one of its inhibitors. Therefore further studies are necessary to elucidate the genuine suppressive mechanisms of MSCs and whether or not they have any relationship with the regulation of granzyme B as presented in ESCs.

## Immunosuppressive Mechanisms of Granzyme B

### Activation Induced Cell Death of T Lymphocytes

Activation-induced cell death (AICD) is a regulatory program co-opted for maintaining the population of activated T lymphocytes induced by repeated stimulation of TCRs (59, 71).

It had been widely accepted that AICD was mediated through the Fas-Fas ligand death pathway until recent literature found granzyme B could promote such process in patients with nonfunctioning Fas (1). Further investigations unveiled a relationship between GrB-induced AICD and the degradation of T cell zeta-chain, a critical component of TCR complex that works with TCR and CD3 molecules to activate both cytotoxic T cells, T helper cells and NK cells (72). Tregs, pDCs and Bregs are frequently witnessed with such consequences. While these suppressor cells making contact with effector lymphocytes, their granzyme B could enter into the target cells *via* three potential pathways: a) passes through membrane pores formed by perforin; b) being endocytosed by membrane repair response during perforin-mediated Ca^2+^ influx; c) adsorbes onto the surface of target cell by electrostatic force that triggers endocytosis (2). Thereafter granzyme B could either directly degrade T cell zeta-chain at multiple sites or trigger the caspase cascade to indirectly cleave it, because T cell zeta-chain is a direct substrate for both caspase 3 and granzyme B. Either way it can abrogate the surface expression of TCR, resulting in malfunctioning T cell activation. Considering the predominance of effector T cells in the setting of anti-tumor immunity, AICD is supposed to be a primary cause for granzyme B-mediated immunosuppression.

Within cytotoxic lymphocytes, granzyme B was expressed and stored in a lysosomal granule if being properly stimulated. However, lysosomal membrane permeabilization (LMP) happens in proliferating and activated lymphocytes and leaks granzyme B into the cytosol, especially when host cells encounter excessive stimulation by TCR. Thereafter, serpin proteinase inhibitor 9 (SERPINB9) would counteract with active granzyme B, preventing it from damaging its host. The competition between SERPINB9 and granzyme B determines the destiny of host cells. If granzyme B overwhelms SERPINB9, it would consequently trigger a series of adverse effects such as direct Bid to the mitochondrial membrane as well as activate caspase 3 and other death substrates, thereby executing AICD (73, 74). Hence AICD is like a suicide program hardwired into cytotoxic lymphocytes that contribute to auto-regulatory apoptosis. This is an important mechanism of self-tolerance to control the size of the lymphocyte pool during and after immunological responses (75).

### GrB-Mediated Cell Death in a Paracrine Manner

In addition to AICD-induced “suicide,” granzyme B could be either intentionally secreted to extracellular space or randomly escape from the immunological synapse between cytotoxic lymphocytes and their target cells during immune surveillance. This diffusive granzyme B would adsorb onto the cell membrane of other bystanders and being endocytosed inside the cells by different mechanisms to induce cell death (76, 77). The randomly escaped granzyme B would flow with body fluid and initiate an indiscriminate attack to any cells it makes contact with, leading to the increased inflammatory status, which might facilitate tumor progression (6). In contrast, vectorial granzyme B secretion is programmed under specific stimulation and often conducted in a contact-dependent manner. For instance, granzyme B can be released from Tregs due to prolonged IL-2 stimulation and non-specific TCR signaling and kills target DCs *via* a perforin-dependent way to undermine adaptive immunity. By analyzing the mobility of Tregs and DCs in TDLNs, a positive correlation between the death rate of DCs and their duration of contact with GrB^+^ Tregs was established, further highlighting the contact-dependent killing mode of extracellular granzyme B (78). Paracrine signaling of granzyme B is a “double-edged sword” that contributes to either immunogenic or immunosuppressive responses, which depends on the origin of those granzymes. Both cytotoxic lymphocytes and suppressor cells (including cancer cells) could fight against each other *via* paracrine granzyme B. Thus results from the overall detection of granzyme B are hard to be interpreted and need scrutinization on the components of specific niche where granzyme B is presented.

### Emperitosis

Secreted granzyme B can be taken back up by its host with potential harm, especially if it were trapped in a confined space (79). During immune surveillance, cytotoxic T lymphocytes (CTLs) could be engulfed into the vacuoles of tumor cells where granzyme B was degranulated. Due to the vacuole restriction, granzyme B cannot be transferred to the cytosol of tumor cells, hence being re-uptaken by its host and initiate a suicide-like death. Such cell-in-cell death is termed Emperitosis (80). It occurred in a variety of tumor types and promoted tumor progression in most cases, which could be leveraged to probe the stages of tumor development (81). Ex vivo and *in vitro* experiments revealed that IL-6 could enhance the adsorption between colon cancer cells and CTLs by upregulating the expression of cell adhesion molecule ICAM1 and polarize CTLs into cancer cells through STAT3, STAT5, ERK, and Rho-ROCK signaling pathways, both of which facilitated the formation of cell-in-cell structure. Furthermore, IL-6 could promote the autophagic activity of target cancer cells after engulfing CTLs, so that protect them from toxic effects and help them survive immune surveillance (82, 83). These results suggested a unique mechanism for immune evasion of cancer cells in TME.

### Facilitate Tumor Angiogenesis

As a potent serine protease, granzyme B can modulate the configuration and components of ECM by degrading vital conjunctions and proteins, which releases some proinflammatory cytokines initially inert or sequestered in ECM. These cytokines would then underlie a favorable TME (84). The immunosuppressive effects that granzyme B enforces through ECM degradation and remodeling were suggested in some reports. For example, granzyme B could release VEGF and TGF-β by cleaving a number of glycoproteins, their anchors to ECM, thereby promoting vascular permeability and tumor angiogenesis during chronic inflammation. Such a process is similar to that presented in the case of MMP-2 and MMP-9 induced tumor angiogenesis (85, 86). Besides, extracellular granzyme B could directly degrade IL-1α within ECM into its fragments that favor the chronic inflammatory environment (87). Therefore, targeting granzyme B in ECM could be a promising strategy to attenuate tumor angiogenesis and mitigate the inflammatory response in TME.

### Potential Determinants for the Immunosuppressive Roles of GrB in TME

Granzyme B expression within TME always experiences dynamic variation along with the pathophysiological changes (88). Except for the aforementioned cells that could express active granzyme B for immunosuppressive purposes, many factors potentially involved in switching the tumoricidal/tumorigenic roles of granzyme B, predisposing a specific niche within TME.

At the initiation phase of carcinogenesis, first responders in the immune system such as macrophages and NK cells recognize and eliminate the immunogenic cancer cells. Within this stage, these first arrivals not only play a direct tumoricidal role but also secrete chemokines like CCL5 and X-C Motif Chemokine Ligand (XCL) 1, which, combining with dangerous signals generated from necrotic cancer cells, recruit other active participants to enhance anti-tumor immunity (89). Once cytotoxic NK cells and effector T cells all got involved, a cytokine storm of granzyme B would show up and is presented as a tumoricidal molecule accompanied by perforin (90). However, the first wave of attack from the immune system is often inadequate for eradicating the cancer cell variants that are less immunogenic. These escaped cancer cells would utilize every resource they have to instigate their bystander cells to establish immune tolerance in a way termed as cancer immunoediting (91, 92). Subsequently, suppresser cells, including tumor-associated macrophages (TAMs), tumor-associated neutrophils (TANs), Tregs, Bregs, MDSCs, and pDCs, etc. are assembled in context-dependent manners and intervene with the anti-tumor immunity shaped by effector cells, where granzyme B possesses dual opposing roles depending on the cell source and relative abundance of those cells in TME (88, 93).

Mechanisms regarding the recruitment of potential immunosuppressive cells into TME have been explicitly described elsewhere. However, a comprehensive understanding of the induction and regulation of granzyme B in these cells is yet to be clarified (94). Though crosstalk between GrB-secreting cells and other players in TME is rather complicated considering the individual differences of host immunity and tumor heterogeneity, GrB^+^ Tregs are generally accepted as central suppressor cells to form the immunosuppressive environment (95, 96). Actually, Tregs share some common routes with effector T cells in the production of granzyme B, such as the JAK/STAT pathway (97). Opposing to the tumor-killing nature of effector T cell-derived GrB, high levels of Treg-derived GrB are confirmed to promote tumor growth (2). Since tumor-specific antigens could both recruit effector T cells and promote the activation and proliferation of autologous Tregs in TME, whatever breaks the balance between Tregs and effector T cells would greatly influence the tumor fate (19).

During immunosurveillance, unconventional TCR stimulation, as well as specific costimulatory molecules and cytokines, could drive the differentiation of CD4^+^ Foxp3^−^ conventional T cells into CD4^+^ Foxp3^+^ iTregs with elevated granzyme B level comparing to nTregs (25, 98). Such iTregs can induce NK cell death in a GrB- and perforin-dependent fashion and inhibit the priming of T helper and effector T cells by GrB-mediated cleavage of their T cell zeta chain (5). Besides, they can kill DCs within TDLNs and TME in a contact-dependent way where Tregs recognize tumor-specific antigens presented by class 2 MHC ligand on DCs and release granzyme B/perforin granules to eliminate them. Hence GrB^+^ Tregs could both impair autoimmunity and prevent the onset of DC-mediated adaptive immunity (77).

**Figure 2:**
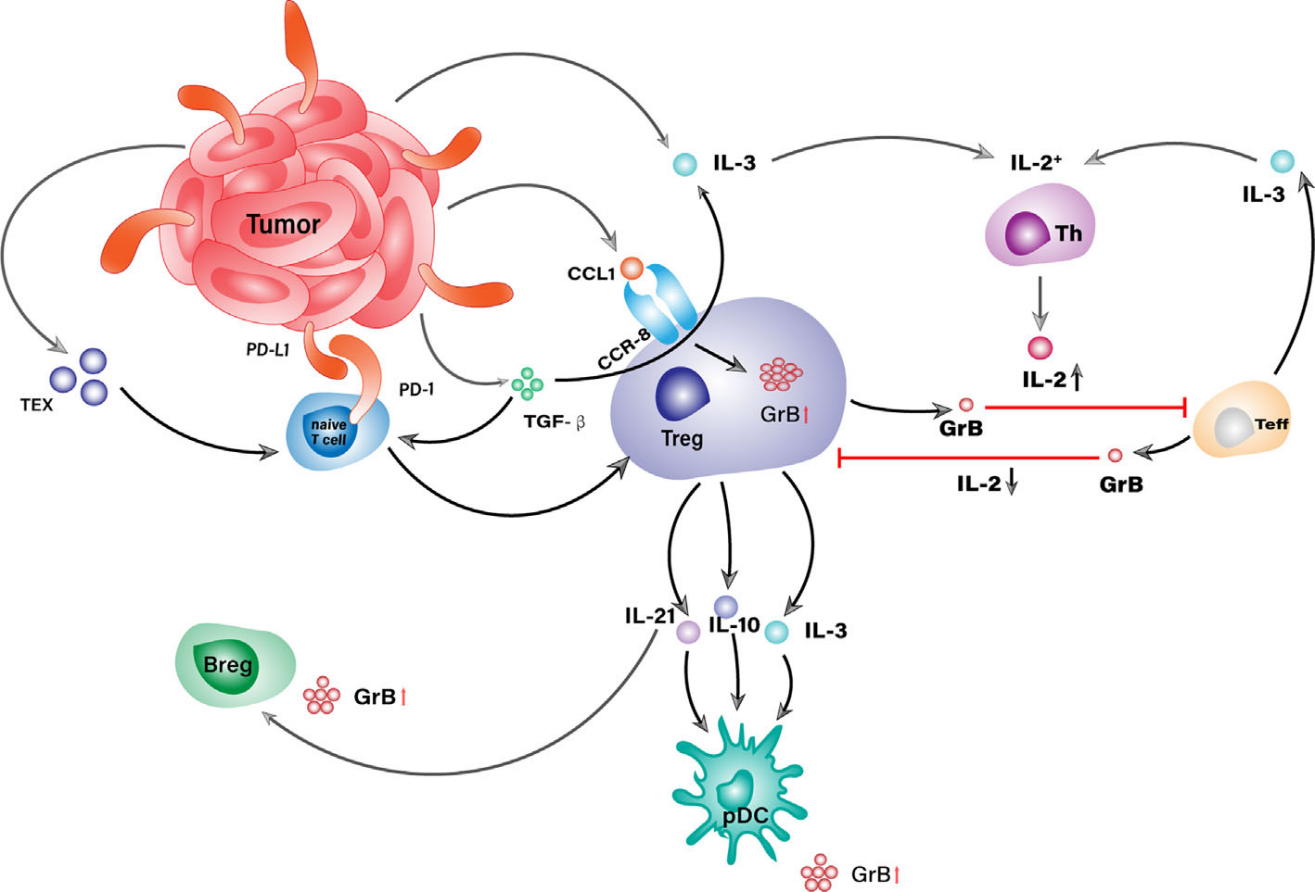
GrB^+^ Treg is a central orchestrator in the GrB-mediated immunosuppressive niche within TME. Tumor cells conjugate with naive T cells *via* PD-L1/PD-1 ligation and convert them into Tregs with the assist of TEX and TGF-β. No matter derived from TME or periphery, Tregs orchestrate the generation of other GrB^+^ suppressor cells as well as the attack against Teffs through the secretion of IL-2, IL-3, and IL-21.

IL-2 has an essential impact on the differentiation and proliferation of both regulatory and effector T cells, hence playing important roles in the tradeoff between anti-tumor tolerance and immunity (99). It could also enhance the expression of granzyme B in both cell types and trigger a GrB-mediated reciprocal death between them, as illustrated in **Figure 2** (100). Detailed investigations of different IL-2 concentrations fed to the co-culture of autologous Tregs and responder T cells (RC) revealed a favorable RC killing toward Tregs under low concentration of IL-2 (150 IU/mL) in opposite to a reverse scenario when its concentration reached 1000 IU/mL. Combined with the fact that IL-2 concentration would experience a phased increase during immune responses, the results above could partially explain T cell exhaustion within TME in the case of malignancies and underline the significance of granzyme B in the setting of immunosuppression (101, 102). Some Tregs, such as Gata3^+^IRF4^+^IL4^+^Foxp3^+^ Th2-like Tregs, are hardwired with enhanced autocrine IL-2-mediated activation so that they could express more granzyme B than other subsets, which helps them survive effector T cells in TME and maintains a tumorigenic environment (103).

IL-3 is another promotive factor for the differentiation of Tregs and often works with IL-2 to facilitate granzyme B expression. After being secreted by activated T cells, monocytes, and/or tumor-associated stromal cells, IL-3 could induce a concomitant increase in the percentage of both Foxp3^+^ Tregs and IL-2 secreting Th cells in a dose-dependent manner (104, 105). The resulting IL-2 would enhance the differentiation of naive T cells into iTregs with high levels of granzyme B. Intriguingly, Tregs could express IL-3 themselves in response to TGF-β, which would further increase the concentration of IL-2 in TME and forms a self-feeding loop to stall anti-tumor immunity (106).

In addition to cytokines, CCL1, a potent chemokine for Treg recruitment in TME, was recently proved to be closely related to granzyme B expression in Tregs (29, 107, 108). It could be secreted by activated monocytes, macrophages, T lymphocytes, endothelial cells, and tumor cells (109, 110). After being released, it can bind with its specific receptor, namely CCR8, on peripheral Tregs and attract them to tumor sites where it induces a STAT3-dependent elevation of granzyme B level that would confer Tregs with a powerful weapon against their targets (29). Other chemokines that could draw Tregs into tumor lesions, such as the ligands for CCR4 and CCR10, are also largely produced in TME, whereas their contribution to granzyme B expression in Tregs is yet to be established (107).

**Figure 3:**
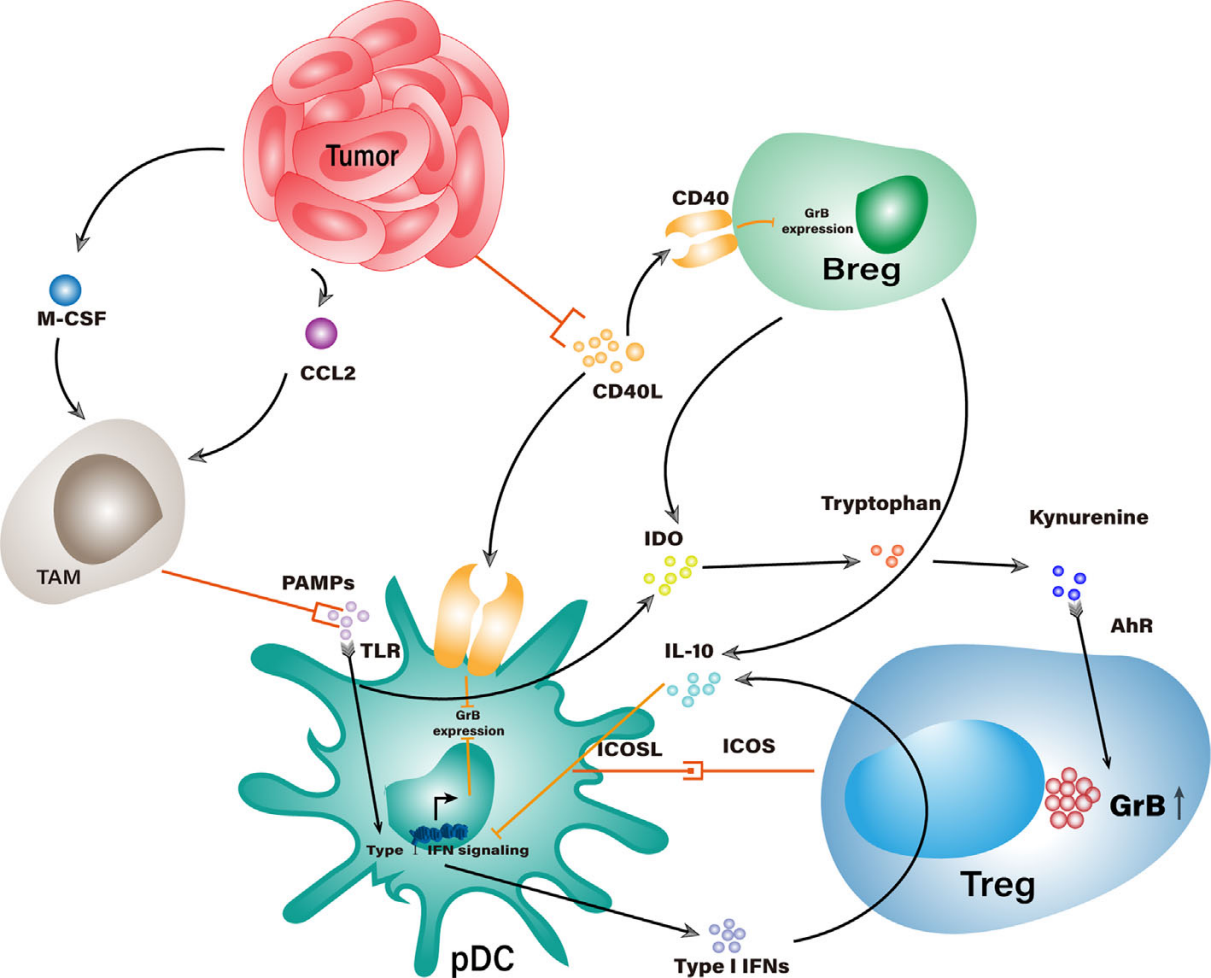
Potential interaction networks that indirectly elevate GrB levels in suppressor cells within TME. Stimulation of TLR would trigger Type I IFN signaling that counteracts with GrB expression in pDCs. Tumor cells recruit TAMs to competitively consume TLR agonists and work with IL-10 from Tregs and Bregs to impair Type I IFN signaling, which indirectly increases the GrB level in pDCs. Tumor cells could consume CD40L and bring down the CD40-CD40L mediated inhibition of GrB expression in Bregs and pDCs. IDO from pDCs and Bregs could enhance GrB expression in Tregs through the catabolite of tryptophan.

Except for secreting granzyme B themselves, Tregs could underlay GrB-mediated suppression in several indirect ways (**Figure 3**). Tumor-infiltrating Tregs were often found in aggregates of other suppressor cells exemplified by TAMs, Bregs, pDCs, and MDSCs, etc. (32, 34). Within the aggregates, Tregs could secrete IL-3, IL-10, and IL-21 that empowers their neighbors with elevated levels of granzyme B. These cytokines are vital factors to granzyme B expression in pDCs, among which IL-21 is the dominant driving force for the generation of GrB^+^ Bregs (8).

However, some immunogenic components could counteract the granzyme B expression in suppressor cells where these cells have been adapted to fight against such adversities. TLR-mediated stimulation is a general response in leukocytes encountering pathogen-associated molecular patterns (PAMPs) derived from cellular components (111). When pDCs recognize PAMPs, the upcoming stimulation will activate their type I IFN signaling pathway and quench the granzyme B expression, thereby converting tolerogenic pDCs into immunogenic pDCs. Interestingly, type I IFNs generated from pDCs could promote IL-10 production in Tregs, which would, in turn, abrogate the type I IFN signaling pathway in pDCs and gradually restore their expression of granzyme B (112, 113). To add fuel to the fire, TLR stimulated pDCs could express the inducible costimulatory molecule (ICOS) ligand, which binds with ICOS on Tregs to promote their expansion and IL-10 secretion (114). Meanwhile, pDC-derived IDO can catabolize surrounding tryptophan into kynurenine derivatives which work on the aryl hydrocarbon receptor (AhR) of Tregs and stabilize their suppressor phenotype with productive granzyme B expression (115–117).

In another respect, IL-21-induced GrB^+^ Bregs contribute to the granzyme B regulation networks within these suppressor cells in similar ways as described above. Put aside whether or not GrB^+^ Bregs could express TGF-β, which still remains to be determined, they are definitely capable of IL-10 and IDO secretion, which positively relate to granzyme B production in both Tregs and pDCs (32, 118). Some components in TME, even not directly linked with granzyme B expression, can skew the immune homeostasis in favor of suppressor cells, facilitating the GrB-mediated immunosuppressive responses. CD40 and CD40 ligand (CD40L) are pivotal costimulatory molecules to the licensing of DCs and activation of effect T cells (119–121). Suppressor cells such as pDCs and Bregs also express CD40 and interact with CD40L on effector cells (122, 123). Unfortunately, CD40-CD40L interaction between regulatory and effector cells is most likely detrimental to granzyme B expression in the regulatory types and even transforms them into tumoricidal cells (34, 51). But that does not stop tumor cells from fighting a way out from their demise. Some neoplastic cells constitutively express CD40 and competitively consume CD40L from activated T cells, thus protecting granzyme B-expressing regulatory cells from turning anergy (124, 125).

Tumor-derived exosomes (TEX) are another powerful weapon tumor bears to inhibit the proliferation and viability of multiple immune effector cells. Researches indicated that exosomes from either the *in vitro* culture of tumor cells or the peripheral blood of tumor-bearing patients could educate CD4^+^CD25^-^ T cell into iTregs with elevated granzyme B level, which effectively suppressed the immune responses against tumors (126, 127). But the understanding of what in TEX and how these components elicit such effect keeps limited.

In contrast, TGF-β is a common regulatory factor in immune response and could explicitly increase granzyme B level in Tregs (128). Tumor cells, TAMs, tumor-associated neutrophils (TANs), Tregs, and MDSCs generally secrete TGF-β while express surface PD-L1 that binds with PD-1 on T cells, which all together promote the expression of FoxP3, thus differentiating T cells into GrB^high^ iTregs (129).

Tumors in both mice and humans secrete high levels of macrophage colony-stimulating factor (M-CSF) and CCL2, potent chemoattractants that could recruit macrophages to tumor sites where they would be educated into TAMs (130, 131). In malignant tumors, TAMs are the most densely populated cell type among all white blood cells, therefore deemed as the major driving force for TME formation (132). Other than direct suppression on T cell function through the surface presentation of several immunosuppressive ligands, TAMs are an abundant source of IL-10 and TGF-β, both of which crosslink with the regulation network of granzyme B and might boost its levels in Tregs, Bregs, and pDCs (133). Although they act aggressively in ingesting tumor antigens, they have been proved relatively inert to trigger adaptive immunity in contrast to effector DCs (134). Given the high density of TAMs in TME, they are speculated to consume most of the immunogenic cellular segments, including TLR agonists, so that might alleviate TLR-mediated granzyme B reduction in pDCs.

The last concern regarding the immunosuppressive roles of granzyme B might focus on its attack mode against effector cells. Since cell-to-cell contact is not necessary to all GrB-mediated immunosuppressive processes, one may wonder if the secreted granzyme B would escape from its original mission and harm adjacent tumor cells instead. Theoretically, the ubiquitous secretion of granzyme B into extracellular space is somehow harmful to every cell that it gets in contact with. That might explain the contradictory discoveries on some GrB-expressing suppressor cells that also pose threats to tumor cells in a GrB-dependent manner (135–137). Among the strategies that tumor cells employ to survive the adversities caused by effector lymphocytes, the one for overcoming GrB-mediated apoptosis is unique and convergent on SERPINB9, the well-defined granzyme B inhibitor that protects its host from being killed by this cytotoxic molecule (138, 139). While upregulation of SERPINB9 has been observed in several tumor types and linked with their resistance to T cell-mediated killing, a comprehensive understanding of SERPINB9 regulation in tumor cells within TME remains in the dark (140–143).

Some researchers confirmed increasing concentrations of estrogen, as well as elevated expression of estrogen receptor alpha (ERα), significantly elevated SERPINB9 level in breast cancer cells, thus effectively deactivated granzyme B and mitigated NK cell-induced cell death. Such effect might be tissue- and/or cell-line-specific due to the causation between estrogen and breast cancer, which is doubtful if it could apply to the increased expression of SERPINB9 in other cancers (144). Another meaningful discovery unearthed that type I IFNs could upregulate SERPINB9 in certain cancer cells, thereby blocking GrB-mediated apoptosis and leading to a subsequent insusceptibility to T cell killing after radiotherapy. Since type I IFNs are frequent participants in TME and most cancer cells express their receptors, induction of SERPINB9 should be a more plausible mechanism underlying the evasion of tumor cells from GrB-dependent proteolysis (145).

## Conclusion

Though granzyme B demonstrates pleiotropic effects in different hosts, excessive expression of granzyme B within the same context has been proved to culminate in anti-tumor propensity due to its innate cytotoxicity (89). In many cases, the GrB-mediated immunosuppression had been treated as collateral events to the interaction between the immune system and pathogens. However, if we zoom in on the battlefield of tumor immune microenvironment (TIME), granzyme B could be evidenced in most fights. The truth is we focused too much on the tumoricidal effects of granzyme B and somehow neglected what it can do to other participants in immunosurveillance. In this review, we introduced several suppressor cells that could secrete active granzyme B for immunosuppressive purposes and discussed possible mechanisms involved in the occurrence of such effects based on what has been documented. Cells with a sporadical expression of granzyme B were not included because little is known about what they utilize granzyme B for. Besides, the gene expression of granzyme B in some suppressor cells is not parallel to the actual level of secreted protein, suggesting the involvement of post-transcriptional regulation (146, 147). All we know now is the signaling pathways of granzyme B in suppressor cells are similar to those seen in effector cells, which converged on the transcription factors of JAK1, STAT3, and STAT5 (8, 148). Recent studies demonstrated that JunB, the AP-1 transcription factor, was essential to the differentiation of effector Tregs and the expression of their effector molecules, including granzyme B (149, 150). But that study did not go deep into detailed investigations of regulation networks around granzyme B. Another noteworthy issue is some tumor cells are observed with endogenous granzyme B and suspect of expressing such proteinase themselves. Nonetheless, the reason why tumor cells evolved to produce granzyme B, which might lead to their suicide, is still unknown (151, 152). Further researches are encouraged to address these issues and should include the GrB^+^ suppressor cells developed from all organs of the immune system for a comprehensive understanding of the immunosuppressive roles of granzyme B.

## Author Contributions

WW wrote the manuscript. TH and ZQ conceived the review and designed the figures. RZ helped with the literature and polish the manuscript. YQ, JL, and YX gleaned the material and scruntinized the manuscript. All authors contributed to the article and approved the submitted version.

## Funding

This research was funded by National Natural Science Foundation of China (Grant No. 81703643 and No.81973712).

## Conflict of Interest

The authors declare that the research was conducted in the absence of any commercial or financial relationships that could be construed as a potential conflict of interest.
